# Do women, men, and companies related to economics use X in the same way?

**DOI:** 10.1016/j.heliyon.2024.e40864

**Published:** 2024-12-03

**Authors:** Marcos Antón-Renart, Esther Ortiz-Martínez, Salvador Marín-Hernández

**Affiliations:** University of Murcia. Department of Accounting and Finance, Murcia, Spain

**Keywords:** Social networks, X, Twitter, Economics, Gender, Behavior

## Abstract

The unquestionable importance of social networks as a means of communication in the 21st century leads us to analyze the behavior of a sample of X accounts representing people and firms who have shown a link with the world of economics at an international level. We analyze these agents in-depth and their behavior in this social network. We conclude that significant differences exist in how women, men, and companies interact on X. The information the accounts provide in their biography and the most used hashtag is also revealing. The results show some tips that will help use an X account, depending on whether the user is legal or natural. They show the reality of their accounts and how they use them. These results are helpful for professionals and policymakers as a departure point for looking for solutions that can be learned from others to increase the popularity or impact of their accounts.

## 1 Introduction

The generalization of the use of the Internet has led to the possibility of using social networks as a means of communication, standing out as an advantage of mass participation and collective intelligence [1]. Previous analyses focus on X (formerly Twitter) within these recent mass communication channels. Specifically, X allows tracking the activity of the users’ accounts to analyze their interactions [2]. X has been a revolution in all areas of information disclosure, and the economy is one of them. In the business world, the need to have a profile in this social network [3] has also been extended as a language to all stakeholders interested in the company, whether natural or legal. Previous studies also demonstrated that females and males manage their social networks substantially differently [4].

In this research, we will focus on a unique group of individuals and entities whose profiles clearly demonstrate a connection to economists or economics. By analyzing their profiles and interactions on X, we aim to address a series of research questions. This study is significant as it contributes to the existing literature on the use of X, a field that has seen limited exploration in the context of economics professionals.

Our study delves deeper by examining specificities in the use of X, depending on whether the account is a legal entity (company or institution) or a natural person (man or woman). The results provide practical tips for managing an X account, as they reveal significant differences in how people interact on X based on their legal or natural status. These findings not only enhance our understanding of social network behavior but also offer actionable insights for professionals, practitioners, and policymakers, empowering them to enhance the popularity or impact of their accounts.

## 2 Theoretical background

Regarding the business sector, or that of the economy, which is the one that concerns us, there is previous research on X that addresses two perspectives: a) that of the company as a transmitter of information [3]. For instance, companies should make special efforts to engage stakeholders in their Corporate social responsibility (CSR) communication on X [5], and b) that of the CEOs, who also try to satisfy the interest groups through X [6].

X implies a technical system for a community because there must be union points and shared objectives when using it [7]. The links can be different; in our case, we focus on economists and/or economics as an essential field of research. This focus is justified as Holmberg and Thelwall [8] check that there are differences in how X is used depending on disciplines and one analyzed in economics. Their results highlight that sharing links or retweets depends on the discipline. In this sense, researchers in economics share more links than researchers in digital humanities and cognitive science. Also, the scientific use of Twitter in economics, sociology and history is lower than in biochemistry, astrophysics, cheminformatics and digital humanities [8].

Using social media is different from being an individual or a collective. Aladwani [9], analyzing data from 41 Twitter accounts and primary data from 20 interviewees, as well as survey responses from 58 individual, business, and government participants, asked why individuals, businesses, and governments would use X, identifying three possible reasons: engagement, access to people, and word of mouth. The findings revealed that individual accounts led the other groups in all three measures. Surprisingly, however, business accounts ranked last in their ability to generate retweets.

Obschonka et al. [10] check these differences for entrepreneurial behavior, arguing that in the case of individuals, there are factors such as education, experience, age, or even gender that determine this behavior. At the same time, collective behavior shows other patterns; in our case, it is studied for legal persons. X, as a database of behavior patterns, has even been used as a predictive system [11].

Page [12] establishes differences between natural and legal persons, concluding that the latter do not personalize their messages using conventional media communication tools.

Therefore, having personal information to analyze makes it possible to observe apparent differences in the use of social networks depending on the user's gender. Alvídrez and Franco-Rodríguez [13] conclude that social context and cultural factors influence text messages, according to whether a man or a woman writes them and condition the style used. Gender differences in teacher X chats are also demonstrated [14], evidencing that men and women tend to participate differently in teacher X chat spaces. Ikae and Savoy [15] also evidence a gender distinction on X, finding stylistic variations between men and women. Differences in the topic of the tweets are also analyzed and found on X between women and men [16,17]. What interacts more in other social networks and how is also analyzed by Hayat et al. [18], finding differences between women and men again. Also, differences are found in the continuance intentions of social network users and how this is gender-sensitive [19]. Gender creates a bias that can lead to misgendering X and reinforcing social prejudices [20].

Also, data on the X usage was analyzed in terms of age, gender, and ethnicity by Longley et al. [21], who found that Males tweet more than Females during the week, including weekends. The specificities of using X created by gender have made several studies infer the gender of the users analyzing the freely available data on X [22,23].

The background mentioned leads us to address the first research question, bearing in mind differences depending on natural or legal persons using X and gender within natural persons:

RQ1 Do women, men (natural persons), and companies (legal persons) related to economics act differently on X?

Regardless of their form and size, legal persons use X as a social media to disclose, and corporate reporting is based initially on agency theory [24]. Afterward, this theory evolves into the stakeholder and legitimacy theory [25,26]. Legal persons disclose seeking legitimacy from stakeholders on the undertakings' practices [27,28]. X is another tool to succeed, although sometimes significant differences exist between the information interests of companies and stakeholders [29]. Therefore, disclosure through X by legal persons may differ from individuals, as the goals are different. Factors such as size determine this corporative disclosure, as large companies have more powerful stakeholders to report [30]. Also, large companies, or even Governments [31], must send a sign of credibility to the markets [26] as has been checked a positive capital market impact of traditional disclosure [32,33] that could be extended to X.

The institutional theory could also be cited, as institutional pressures play a vital role in explaining the behaviors of companies when engaging in non-financial or sustainability disclosure [34]. The institutional theory even suggests that all organizations tend to behave similarly [35], whether due to external coercive pressures (such as mandatory regulations), normative pressures (such as accepted or assumed values or principles), or even the imitation effect [36,37].

Another argument that is also valid for the use of X to disclose information by legal persons is the positive effect on the company's reputation [38,39]. Internal and external legitimacy could trigger disclosure in X to respond to all pressures, as with traditional reporting channels [40,41]. Nevertheless, these legitimacies can differ depending on an individual's or a corporation's message, the former based on internal legitimacy and the latter on external. As Van der Steen et al. [42] remark, there is an important stream of academic work exploring the ways to get external legitimacy, while there is a more limited understanding of how to acquire internal legitimacy, which internal stakeholders confer when there is an evaluation by internal audiences. Although the differences on X, depending on whether the user is a natural or legal person, are explained, all the specific key characteristics of the accounts and the way users tweet, which are analyzed in this paper, interact together, and they can be done in a layered way or with a neutral result. Therefore, checking these features' interactions is also necessary because, as Orellana-Rodriguez [43] points out, "very few controlled studies have specifically examined the interactions between these variables." Hence, all this previous literature takes us to propose our second research question:

RQ2 Is there a significant relationship between the main features of these types of accounts?

A way to measure the popularity and social influence is the Klout analysis for these accounts, which can also be related to a greater power or leadership that these users want to transmit since previous research shows a close relationship between both [44]. The Klout index has been very popular internationally for years. It measures the degree of influence of a person or brand on social networks, giving a score from 1 to 100. The higher the mark, the better the degree of influence. Considered the most sophisticated and popular indicator, Klout scores are based on true reach, amplification, and network impact [45]. Other ways to measure popularity and social influence can be seen in Garcia et al. [46].

However, the Klout analysis and the total number of tweets depend on the years of existence of the X account. Therefore, a background introduces the time dimension as the year of publication, which we consider as the age of the account. It captures the effect of the time trend. Zhang and Wang [47] obtain that time matters, and it is one of the two most relevant factors determining popularity. However, there are other views to predict the time needed to reach its popularity peak [48] or different times as popularity varies over time [49]. Regardless of the time measurement, there is a relationship between both variables. Therefore, according to this background, we establish our third research question:

RQ3 Is the popularity of the account related to its age?

Other research focuses on cultural, social, and language factors influencing and determining the communication process, such as the study of the most popular languages used on X by Hong et al. [50]. Also, geographical origin determines cultural values to a large extent and has thus also been analyzed [51,52]. García Molina and Chicaíza [53] even affirm that the campaigns′ results on three topics of great international relevance are not only affected by economic issues but also by cultural factors.

As a source of information, the background research uses the profiles of the users and their way of interacting and analyzing their habits and routines [52,[54], [55], [56]].

The economists′ use of social networks as a professional profile may be related to the role of CEOs and their activity on X [6] or the image of companies through the publication of information [3].

In any case, some analyses claim a positive relationship between interactions in social networks and users of the same profession [56]. For example, Pentina et al. [57] conclude that origin does not condition that professionals use X as a tool for professional development.

An essential line of research studies the relationship between user bios and X. This link is so narrow that the analysis of bios can predict the linkages between X users [58]. In addition, analyzing tweets, retweets, and user list memberships can describe the X user's expertise [59]. These previous studies use quantitative analysis to establish differences and similarities between the profiles using tweets or hashtags [[60], [61], [62]], and others add qualitative analysis, such as Muthusami and Bharathi [63]. Ding and Jiang [64] discovered that bios are a complementary source of information for analyzing tweets and that profiles have a significant link with discovering quality topics. Rogers and Jones [65] establish a relationship between the phrases and the X users' bios. Jones [66] studies X bios and finds that the average American user progressively incorporates politics into their social identity. Moreover, Pathak et al. [67] explore socioeconomic ethnicity derived from X bios. The relevance of X in other economics activities, such as tourism, and its most frequent words can be seen in Curlin et al. [68].

The previous studies show a link between tweets and the bio or profile of X users, which justifies the last proposed research question:

RQ4 How does the information these accounts reveal in their Bio and tweets differ?

## 3 Materials and methods

To compile the data, we have used convenience sampling, as in previous literature about X and other professional aspects, such as Holmberg and Thelwall [8]. In social network-oriented research, it is common to use convenience sampling instead of random sampling. In our case, the population to study is all economic professionals, including the most influential economic influencers on X.

This information was collected over a few months to create a broad database, allowing us to conduct future studies. Hence, we collected the information from April to June 2017, searching with the search engine of the social network X, identifying agents who had included the term economist and/or economics in Spanish (economista and/or economía) in the biographies of their profiles (bio). In addition, using these identified accounts, we looked for their followers and followings like other authors [8] to identify the accounts of the economy professionals. Searching for economics-related accounts through X's search engine (Twitter) led to limited results. For this reason, the results showed a random sample of cases, all those offered by the search engine but not all the accounts linked to the economy. Therefore, if an account was not found using the search function on the X website, it does mean that the existing accounts in X linked to the economy are more than those that appear to you when you search in your search engine. This led us to look for other accounts, in a complementary manner, in other sources of information to broaden the sample, as in Porter et al. [69], a Google search was conducted. This also leads us to find and include accounts of X users registered in the economic activity and economists in the TopInfluencers account. TopInfluencers is a portal that identifies those accounts with the most impact on X through an algorithm. Therefore, the sample analyzed in this paper includes natural persons (NP) (distinguishing between men (M) and women (W)) and legal persons (LP) who claim to be related to the economy or economists.

Thus, information was first collected by searching with the search engine of social network X, identifying agents who had included the term economist and/or economics in their profile biographies (bio). We also considered the agents registered in the field of economics who had the greatest impact on the X social network.

The result was 278 accounts (158 directly from X and 120 from TopInfluencers, excluding overlaps). From these 278 accounts, 156 natural persons, 120 legal entities, and two not identified in the system from 21 countries. In addition, we have included nine cases whose origin is not identified in the study and classified as "others."

Table 1 also shows the total number of cases included in the sample by country and the percentage they represent. Pearson's Chi-square statistical tests were conducted to verify no bias in the sample by agent (natural person/legal entity) and origin (by countries and continents).

**Table 1:** Sample distribution.

COUNTRY	LP	NP	M	W	CASES	%	COUNTRY	LP	NP	M	W	CASES	%
Argentina	9	13	13	–	22	7.9	Italy	4	1	1	–	5	1.8
Austria	1	0	–	–	1	0.4	Mexico	5	7	7	–	12	4.3
Bolivia	1	0	–	–	1	0.4	Nicaragua	2	0	–	–	2	0.7
Brazil	14	8	8	–	22	7.9	Panama	1	1	1	–	2	0.7
Chile	2	4	4	–	6	2.1	Paraguay	0	1	1	–	1	0.4
China	0	1	1	–	1	0.4	Peru	1	4	4	–	5	1.8
Colombia	1	6	4	2	7	2.5	Portugal	0	1	–	1	1	0.4
Cuba	1	0	–	–	1	0.4	Sweden	0	1	–	1	1	0.4
Ecuador	1	1	1	–	2	0.7	USA	1	5	5	–	6	2.1
Spain	70	91	80	11	161	57.9	Venezuela	1	6	6	–	8	2.9
United Kingdom	0	2	2	–	2	0.7	Others	5	3	3	–	9	3.2

Another advantage of our work is that convenience sampling makes our sample of X accounts more extensive than others studied in previous papers [70,71].

To answer the research questions, we have created a database with personal variables obtained from X accounts and X-related variables. All these variables are included in Table 2, Table 3 with their description and the related research question.

**Table 2:** Personal variables included in the database.

Variable	Description	Research question
Legal persons	The X account belongs to a legal person. Therefore, it is a corporative or collective account.	RQ1, RQ2, RQ3, RQ4
Natural persons	The X account belongs to a natural person.	RQ1, RQ2, RQ3, RQ4
Gender	In natural persons′ accounts, if they belong to women or men.	RQ1, RQ2, RQ3, RQ4
Age	When the account was created (in months).	RQ3
Profile photo	Formal; informal; logo; other.	RQ4

**Table 3:** X-related variables included in the database.

Variable	Description	Research question
Following	Number of accounts followed.	RQ1, RQ2, RQ3
Followers	People (NP&LP) following the analyzed account.	RQ1, RQ2, RQ3
Tweets	Total number of tweets published by the account since its inception.	RQ1, RQ2, RQ3
Links in the tweets(%)	% of tweets which include at least one link to a website.	RQ1, RQ2, RQ3
Tweets/day	Number of tweets published by the account daily (including retweets).	RQ1, RQ2, RQ3
Tweets are RTs (%)	% of tweets retweeted of other accounts.	RQ1, RQ2, RQ3
Tweets are answers (%)	% of tweets which are responses to other tweets (usually from other accounts).	RQ1, RQ2, RQ3
Tweets retweeted by others (%)	% of tweets that were retweeted by other accounts.	RQ1, RQ2, RQ3
Times RT	Total number of retweets received in the analyzed period.	RQ1, RQ2, RQ3
Times Like	Total number of Likes received along the period under review.	RQ1, RQ2, RQ3
Own lists	Number of public lists that the account shows.	RQ1, RQ2, RQ3
Other lists	Number of lists where the account was included.	RQ1, RQ2, RQ3
Klout	Klout number (influence of the account in the social networks).	RQ1, RQ2, RQ3
# most used	The hashtag most used by each account.	RQ4
Account impact	(times RT + times Like)/(30xtweets/day)	RQ1, RQ2, RQ3

The content of these variables is taken directly from the account of each analyzed case. Using other tools such as Tweetchup (later Postchup), Klout, and top influencers allowed us to complete the necessary information for our analysis. The statistical analysis was performed with SPSS 28.0 for Windows and R (WRS2). We obtained the cases in each category and the corresponding percentages for the qualitative variables. To analyze the differences between natural and legal persons when interacting on X, we have applied the function that implements the Yuen test for heteroscedasticity [72] because it is not advisable to use the *t*-test to analyze the equality of means when dealing with very asymmetric populations with heavy tails [73]. Therefore, the most advisable alternative uses tests based on robust localization measures, such as trimmed mean. We have also obtained the correlations (robust) at global and individual levels for each X account. Finally, we have applied the Pearson Chi-square test to check the independence between qualitative variables. The accepted threshold for statistical significance is set at p < 0.1.

## 4 Results

### 4.1 Legal and natural person owners of X accounts and their behavior on X. Differences between men and women within natural persons (Research questions 1 and 2, RQ1 and RQ2)

In order to answer the first research question (RQ1), we analyze if there are significant differences between natural and legal persons when interacting on X, and all of them belong to the same discipline: economics, which determines the use of X [8]. The results in Table 4.1 show statistically significant differences between natural and legal entities in the number of accounts they follow (following). There are also differences in the tweets that are RTs, the number of times the tweets are RT and Liked, the impact of the accounts, and the links within the tweets. Finally, significant differences are found in the tweets that are replied to, in the tweets that are retweeted by others, and in the ratio of Followers/Following. Similar but not equal happens when comparing men and women (Table 4.2). In addition, significant differences are found in the number of accounts they follow and their number of followers. The same happens with the number of tweets they usually publish, their lists, the ones they are included, their Likes received, and the percentage of tweets retweeted by other accounts.

**Table 4.1:** Results of the Yuen test for natural and legal entities.

	Natural person			Legal person			Yuen statistic (Sig)
	Mean	Median	Trimmed mean to 20 %	Average	Median	Trimmed mean to 20 %	
Following	6875.86	809	863.87	2496.28	361	454.94	3.457 (0.000)∗∗∗
Followers	62,922.51	7461	12,405.84	69,865.62	9472	13,141.74	0.199 (0.842)
Followers/Following	200.22	8	16.59	2365.98	21	85.04	1.829 (0.072)∗
Tweets	32,690.26	11,202	12,879.15	29,343.09	14,139	17,798.76	1.586 (0.115)
Other Lists	680.64	232	278.42	902.95	198	306.25	0.422 (0.674)
Own Lists	14.21	1	1.60	3.60	1	1.37	0.406 (0.685)
Tweets/day	7.11	4.8	4.94	12	5.0	6.85	1.467 (0.146)
Tweets are RTs (%)	34.27 %	32.65 %	30.77 %	21.20 %	4.95 %	10.56 %	4.717 (0.000)∗∗∗
Times RT	67,457.19	4351.5	8315.218	6539.32	457.5	672.95	3.124 (0.003)∗∗
Times Like	5281.07	353	674.15	1256.95	110	180.87	2.783 (0.007)∗
Tweets are answers (%)	15 %	6.55 %	9.91 %	2.77 %	0 %	0.41 %	5.693 (0.000)∗∗∗
Tweets retweeted by others (%)	73.20 %	81.40 %	78.95 %	64.31 %	74.45 %	68.35 %	2.107 (0.038)∗∗
Links in the tweets (%)	31.19 %	28.94 %	26.66 %	59.26 %	77.27 %	65.20 %	4.849 (0.000)∗∗∗
Account Impact	285.59	55.79	71.12	17.99	4.76	5.17	5.294 (0.000)∗∗∗

Statistically significant at confidence levels of ∗ p < 0.1 ∗∗p < 0.05 ∗∗∗p < 0.01.

**Table 4.2:** Results of the Yuen test for men and women (natural entities).

	Men			Women			Yuen statistic(Sig)
	Mean	Median	Trimmed mean to 20 %	Average	Median	Trimmed mean to 20 %	
Following	7579.31	888	1414.61	450,93	325	437,31	31.43 (0.000)∗∗∗
Followers	68,946.28	7956	35,922.68	7905.47	3446	6887.02	13.62 (0.000)∗∗∗
Followers/Following	219.39	8.48	62.15	25.12	4.28	21.62	8.28 (0.005)∗∗∗
Tweets	34,689.35	11,356	20,160.44	14,431.87	6097	12,096.74	5.39 (0.023)∗∗
Other Lists	637.20	245	470.59	222.46	154	178.07	8.36 (0.006)∗∗∗
Own Lists	15.97	1	3.56	2.31	1	1.40	2.80 (0.097)∗
Tweets/day	7.31	4.80	6.16	5.05	2.95	4.68	1.10 (0.310)
Tweets are RTs (%)	34.00 %	32.10 %	32.45 %	37.05 %	41.00 %	36.63 %	0.03 (0.874)
Times RT	68,288.97	4283	23,049.08	58,610.10	5549	45,897.71	0.08 (0.779)
Times Like	5565.20	388	2304.45	2259.00	149	1753.83	2.97 (0.090)∗
Tweets are answers (%)	14.75 %	6.06 %	12.84 %	17.72 %	2.70 %	16.65 %	0.14 (0.717)
Tweets retweeted by others (%)	73.26 %	81.00 %	75.62 %	72.51 %	83.30 %	73.48 %	2.107 (0.038)∗∗
Links in the tweets (%)	31.08 %	28.94 %	29.18 %	32.32 %	25.41 %	31.14 %	0.01 (0.939)
Account Impact	283.10	55.03	131.15	311.85	88.87	287.10	0.01 (0.929)

Statistically significant at confidence levels of ∗ p < 0.1 ∗∗p < 0.05 ∗∗∗p < 0.01.

Hence, natural persons follow twice as many accounts as legal persons. The analysis of natural persons shows that women follow one-third of the number of accounts men follow. In addition, the variable Tweets that are RTs have shown differences between RTs and other accounts made by natural persons compared to those made by legal persons. From a practical point of view, legal persons usually publish more tweets of their content than retweets to other accounts. However, no significant differences between tweets and retweets by men and women are found.

The number of times the tweets are RT is much higher in the natural accounts than in the legal ones, and, in particular, women use RT more than men, although the difference is not significant. The same occurs for those marked as Like, where natural persons get a more significant number of likes in their tweets than legal persons.

On the other hand, differences are found between men and women, as the likes received by men are higher than the ones obtained by women. These results are obtained from the Yuen statistic, which can show significant differences. As previously highlighted, it can be seen through simulation that the Yuen test controls the Type I error rate for asymmetric heavy-tailed distributions with high heteroscedasticity, even with extremely unequal sample sizes, where one of the groups is quite small, such as 141 and 15. It is worth remembering that a retweet causes the tweet to reach more people (because its scope is greater) and allows the tweet to be seen by the followers of the retweeting account.

There are also significant differences when interacting with followers, as natural accounts reply more to other tweets than legal ones. Nevertheless, no significant differences are found between men and women when replying to other accounts. In addition, there are differences in the percentage of tweets retweeted by other accounts between natural and legal entities as well as for men and women. Of the total tweets published over a period, followers retweet more those of natural persons than legal entities (75 % approximately for men and women). That is, there is a more significant number of tweets that were RT at least once in the case of natural persons than legal persons.

There are also significant differences between natural and legal persons in the percentages of tweets that contain links, which are much higher in legal persons than in natural persons (no significant differences were found between men and women). Legal persons use a link to the information contained in the tweet (in 65.20 % of tweets) and, therefore, reveal more information.

Moreover, the results present significant differences between men and women for the variables followers, followers/following, total tweets, lists to which the accounts belong, and their lists. According to these results, women follow fewer accounts than men and have fewer accounts following them in a proportion of approximately 1/6. As a result of the above, the ratio of followers/following favors men, evidence that women publish fewer tweets than men.

Furthermore, there is a significant difference in the lists. In both cases, other lists and own lists, men are included in (and also create) more than women. Regarding the daily tweets, women publish fewer tweets daily than men, although this difference is not significant. On the other hand, the results do not show significant differences between natural and legal entities for the variables followers, total tweets and tweets published per day, lists to which the accounts belong, and their lists.

Although we see significant differences in the number of accounts followed by natural and legal companies before, the same does not happen with the number of followers, where there are no significant differences. However, both types follow fewer accounts than they have followers, which is a common practice on X. Accounts with many followers do not usually follow accounts with just a few. In addition, lists on X (public and private) allow one to classify and group other accounts, making it easier to find them quickly and monitor them, know what they say, and without the need to become officially "in fact" a follower.

Regarding the total number of tweets published, both groups have published, on average, more than 10,000 tweets since their accounts were created, implying they are active in this social network. No significant differences are found in the variable tweets per day, with both groups publishing a similar number. However, they do not reveal the same amount of information if we look at the content.

As for the lists that the account belongs to, we observe that accounts are included, on average, in more than 250 lists (278 for natural persons and 306 for legal persons). One way to measure an account's degree of influence is the number of lists in which an account is included. These lists are few on average and fewer than the number of lists in which they are included.

Therefore, it has been checked that natural and legal persons behave differently on X, as concluded by Obschonka et al. [10] about the use of social media. The account owner determines the X action, so the first research question (RQ1) is answered.

Table 5 includes the relationship between the main features of accounts depending on the account owner (whether natural or legal persons and within natural persons, whether men or women).

**Table 5:** Variables correlation: Natural (N); Legal (L); Men (M) and Women (W).

Global Level-N&L Pearson correlation	Other Lists	Own Lists	Klout	Followers	Account Impact	Age
Other Lists	1					
Own Lists	0,195∗∗	1				
Klout	0,545∗∗∗	0,087	1			
Followers	0,670∗∗∗	0,143∗	0,465∗∗∗	1		
Account Impact	0,123∗	0,001	0,213∗∗	0,237∗∗∗	1	
Age	0,342∗∗∗	0,120	0,230∗∗∗	0,255∗∗∗	0,107	1

Legal Person. Pearson correlation	OtherLists	OwnLists	Klout	Followers	Account Impact	Age

Other Lists	1					
Own Lists	0,189∗	1				
Klout	0,510∗∗∗	0,260	1			
Followers	0,714∗∗∗	0,054	0,441∗∗∗	1		
Account Impact	0,057	0,302∗∗	0,153	0,047	1	
Age	0,302∗∗∗	0,15	0,045	0,254∗∗∗	0,113	1

Natural Person-M&W Pearson correlation	OtherLists	OwnLists	Klout	Followers	Account Impact	Age

Other Lists	1					
Own Lists	0,336∗∗∗	1				
Klout	0,654∗∗∗	0,079	1			
Followers	0,617∗∗∗	0,232∗∗	0,504∗∗∗	1		
Account Impact	0,229∗∗∗	−0,026	0,269∗∗	0,354∗∗∗	1	
Age	0,411∗∗∗	0,159	0,393∗∗∗	0,257∗∗∗	0,140	1

Men accounts Pearson correlation	OtherLists	OwnLists	Klout	Followers	Account Impact	Age

Other Lists	1					
Own Lists	0,329∗∗∗	1				
Klout	0,660∗∗∗	0,053	1			
Followers	0,614∗∗∗	0,224∗	0,506∗∗∗	1		
Account Impact	0,237∗∗	−0,032	0,266∗∗	0,360∗∗∗	1	
Age	0,410∗∗∗	0,149	0,395∗∗∗	0,250∗∗∗	0,139	1

Women accounts Pearson correlation	OtherLists	OwnLists	Klout	Followers	Account Impact	Age

Other Lists	1					
Own Lists	0,346	1				
Klout	0,543∗∗	0,559∗∗	1			
Followers	0,773∗∗∗	0,332	0,600∗∗	1		
Account Impact	−0,171	0,644∗∗	0,489∗	0,304	1	
Age	0,355	0,554∗∗	0,12	0,497∗	0,372	1

Statistically significant at confidence levels of ∗ p < 0.1 ∗∗p < 0.05 ∗∗∗p < 0.01.

These results will answer the second research question (RQ2). According to these, we can assert that there are different relationships between the main X variables determined by the type of account. We can highlight the following relationships:

a) Concerning the lists to which the account is included, there is a positive correlation with the number of lists, Klout, followers, impact, and age of the account [47].
b) A positive correlation exists between the number of lists and followers. However, there is no correlation between Klout, impact, and age.
c) There is a positive correlation between the Klout and the lists to which the accounts belong, followers, impact, and age [45]. On the other hand, there is no correlation between the number of lists the account has and its popularity index on social networks [46].
d) There is a positive correlation between the number of followers and the lists in which it is included, including its own list and Klout. Also, there is a correlation between the account's impact and age.

### 4.2 Legal and natural person owners of X accounts and their profiles. The popularity of the accounts and relationship with their age (Research questions 3 and 4, RQ3 and RQ4)

Regarding the impact of the account, there is no correlation between its lists and age. However, there may be a correlation between the account's age and Klout's (Table 5). The index of the popularity of social networks is a reference for calculating the index for three months (as well as the participation of the account in several social networks). Thus, it could be reasonable that accounts opened long ago have a greater Klout. Hence, age positively correlates with all other variables except impact and own lists because although time matters [47] there are other ways to measure it [48,49]. These results answer our third research question (RQ3).

The analysis of the profile on X will allow us to answer research question 4 (RQ4). First, we wonder about the image the accounts want to show with their profile photo and the professional activities they highlight. The results (Table 6) show that half of the natural persons include a formal photo in their profile, while a third offer an informal image. Although eye-catching, we consider a more carefree image can respond to the search for a greater approach to users in this social network. A comparison of men and women shows that nearly 75 % of women choose a formal photo in their profile, while 13.3 % choose an informal image. On the other hand, 46.8 % of men prefer a formal image, while 33.8 % upload an informal photo. As expected, very few individuals post a logo as a profile image (any women and just 2.9 % of men), and 16 % appear with other types of photos (men and women practically the same percentage). The reverse happens with legal entities, with a logo as an image (86 %), Others (11.9 %), and a formal or informal photo as a residual percentage. The results obtained from the Pearson Chi-square test (value 207.87 p-value<0.001) take to the rejection of the independence between the variables Profile photo and Person (natural or legal). Therefore, natural and legal persons use different types of photos, establishing differences in the bios and profiles depending on the account user [58,64,65].

**Table 6:** The profile photo.

	Formal	(%)	Informal	(%)	Logo	(%)	Others	(%)	TOTAL	(%)
Natural person	76	49.4	49	31.8	4	2.6	25	16.2	154	100
Men	65	46.8	47	33.8	4	2.9	23	16.6		
Women	11	73.3	2	13.3	0	0	2	13.3		
Total M − W	76	49.4	49	31.8	4	2.6	25	16.2	154	100
Legal person	2	1.7	1	0.8	101	85.6	14	11.9	118	100
Total N-L	78	28.7	50	18.4	105	38.6	39	14.3	272	100

As for the professional activities the accounts highlight (Table 7), one-third of the sample identifies with the professional activity of economist, followed by communication (journalists, press); other professional activities (16.6 %); teachers and researchers (15.2 %); policy (9 %) and finally Others (1.4 %). Of the total sample, 25.2 % reflect two professional activities, highlighting, in this order, communication; other professional activities, teachers and researchers and economists. X is a tool for professional interactions and a sign of identity for users of the same profession [56,57].

**Table 7:** Professional activities highlighted in the X profile.

	1 activity (%)	2 or more activities (25.2 %) (%)
Professionals	16.6	28.6
Economists	32.4	11.4
Teachers and Researchers	15.2	18.6
Communication	25.5	30.0
Politics	9.0	10.0
Others	1.4	1.4
Total	100.0	100.0

Finally, we consider what natural and legal persons emphasize as keywords in their tweets. We analyze each account's hashtag (#) used most in its tweets (Table 8). Curiously, a quarter of natural persons do not use hashtags, and this percentage increases to 38 % in the case of legal companies, which do not use this option and, therefore, do not highlight any keyword. Elsewhere, we can highlight politics; news in general, stock exchange/finance/accounting, and places to a lesser degree, but hashtagged are references to events that are happening at that moment (#estápasando), sports, and even references to facts, events and news from the past. Moreover, men and women prefer not to use hashtags in their tweets, when they do, they use ones related to politics and places.

**Table 8:** The most used hashtag (#) by the accounts.

Topics	#					# (%)				
	LP	NP	M	W	Total (L + N)	LP	NP	M	W	Total (L + N)
News in general	19	14	13	1	33	15.8	9.0	9,2	6,7	11.9
Anything (blank)	45	39	35	4	86	37.5	25.0	24,8	26,7	30.9
Sports	1	8	8	0	9	0.8	5.1	5,7	0,0	3.2
Is happening	8	8	6	2	16	6.7	5.1	4,3	13,3	5.8
Stock/Finance/Accounting	16	12	11	1	28	13.3	7.7	7,8	6,7	10.1
Looking back	2	1	1	0	3	1.7	0.6	0,7	0,0	1.1
People	4	4	4	0	8	3.3	2.6	2,8	0,0	2.9
Politics	6	35	32	3	41	5.0	22.4	22,7	20,0	14.7
Places	8	20	17	3	28	6.7	12.8	12,1	20,0	10.1
Other (miscellany)	11	15	14	1	26	9.2	9.6	9,9	6,7	9.4
Total	120	156	141	15	278	100	100	100	100	100

## 5 Discussion

From the analysis of a random sample of X accounts that show a sense of belonging to economics and/or economists [8], we conclude that there are significant differences in how people interact on X depending on whether they are natural or legal [10,12] as X can be used as a source of sociological studies [74]. Furthermore, the gender analysis of X users also reveals that men and women use this social network differently.

In practice, natural and legal persons behave differently when following other accounts. Therefore, it is likely due to the application of strict reputational prudence or other reasons that institutions' accounts generally follow fewer accounts than natural persons. In addition, the accounts they follow are usually from other institutions/companies and employees of the company under analysis. The impact of the publications of natural persons is higher than that of legal institutions/firms. That is, their messages reach a more significant number of followers and are viewed more (and usually read); consequently, there are significant differences in the impact of the accounts. All these differences are also the consequence of legal persons′ use of X because they do not personalize their messages using conventional media communication tools [12]. Moreover, this significance needs to be present when comparing the account impact of men and women [[14], [15], [16], [17]].

To sum up, the greater the degree of influence on social networks, the greater the number of lists and followers included in. As obtained, the variables Klout or Account impact show a positive and significant correlation between the variables. Therefore, we can conclude that the greater the accounts′ impact and their degree of influence or popularity, the more people tend to include them in their lists of favorite economic accounts [47,49,75,69]. Hence, the account's impact and age are more significant as time matters [47]. It implies that an account that regularly publishes quality content gains followers over time as there is a correlation between the account's age and Klout's. However, an account that has been active for a long time does not necessarily imply that it has a more significant impact because popularity varies over time [49]. In any case, X is used to increase visibility, be this personal or professional, for the company or company products [75] or to improve personal status or that of the company [69].

Therefore, we find more significant differences between both types of persons, corroborating the results obtained in other works such as Hanusch [76], which find different behavior patterns on X depending on whether the user is a natural or legal person. In addition, the analysis of several correlations shows significant differences, generally, between the performance of an account (natural or legal) and its influence on other accounts. The correlations between Klout influence, account impact, and the number of followers support our case. The metrics obtained in the ratio of followers/following and the times their posts are liked or Retweeted lead us to a robust conclusion, reinforcing the validity of our statement.

The profile analysis of the users shows significant differences in the type of photo that natural and legal persons use [3,6]. They highlight in their profiles the profession of Economist, followed by Communication and professionals. Finally, on the topics repeated as keywords, it is noteworthy to see that in one out of three tweets, the accounts do not indicate any topic and that politics, news in general, and stock market/finance/accounting are the most highlighted issues. In this sense, we can conclude, like Pentina et al. [57], that X is used for the professional development of the collective we analyze, regardless of the country of origin. A further conclusion is that using X is relevant to the tasks these economy professionals can develop [77].

In order to go more deeply into the study of the use of X by professionals related to economics, we consider the future, including the analysis of other variables when finding significant differences. Concretely, among these new variables considered for our future analysis are X-verified accounts, i.e., those with the 'Blue checkmark' or Twitter verification badge, which initially indicates that this is a prominent account because of the notoriety of the person/institution behind it. Another variable that could be analyzed in the future is whether it discourages or encourages the use of social networks based on users' responses to their posts.

In this sense, in the future, we will introduce new hypotheses, checking whether there is a relationship between the interaction received by an account's posts over time and the greater or lesser publication of content by the accounts.

Nowadays, when social networks are extensively used, and X is a mass communication tool on a professional level, the practical implications of this research can be pointed out. Of course, media economists and practitioners have to bear in mind that there are differences in the use of X depending on the type of user, whether natural or legal persons. However, some specificities can be learned from the other to increase the popularity or impact of their accounts. For example, legal persons can increase their impact and change how they manage their accounts, adapting some good practices from individuals. Moreover, at the same time, individuals can increase their visibility more formally using practices from corporate accounts.

The results also show some tips to help use an X account, depending on whether the user is a legal or natural person. Some advice can be taken from the results obtained in the research, as it shows what information economists usually publish in their biographies (quantity and subject matter), the frequency with which they publish, whether they usually include additional information in their tweets such as a link, whether or not they usually follow other accounts, whether they usually publish more posts prepared by them, whether they usually make direct use of RT, whether they usually publicly display lists and therefore, whether they make use of this tool and even whether they make use of hashtags in their publications. Going into detail, we indicate these tips observed from the analyzed variables for better management and success of an X account. First, related to the Account impact and the content of posts (hashtags, links, and others): learn from everyone what works and what does not, what to do and what not to do; see what they talk about and how they do it. This advice is for both NP and LP; Legal Persons should identify the topics and the content of the posts sent in their tweets. They will also decide if they will just post content related to the company/institution's activities and/or they will share the content from other sources; also, for both natural and legal persons, the usage of a hashtag will increase the visibility of a tweet; the reality has shown us the higher use of links made by LP than NP. These seem reasonable, as the possibilities to use them are higher for LP (as it expands its content in its websites, news, …). Regardless of the type of account, it is advisable to include always links in the posts when the information contained in the tweet can be increased using the link. Secondly, about the account followers or who to follow (following): any account should define its policy of following other accounts. LP should be clear about which accounts they will follow and which ones will not. In the case of NPs, they do not have that need, but, as a general rule, it is suggested that they follow or follow back an account just if they feel comfortable and/or think that they find that account interesting. It is also advisable to look at their profile and tweet the timeline of another account before following it; Natural and Legal Persons need an audience. The content of a tweet can be relevant, but nobody will read it if there are no followers. Account verification can bring more visibility to posts; as we have demonstrated, X users prefer to follow fewer people than their number of followers. It is advisable to get it (for both NP and LP); also, for both types of accounts, when responding to a question, comment, or offense, …. whether from a follower or not, first assess what you will say and how.

Regarding abusive tweets or insults, report the tweet to X and recommend blocking the offending or insulting person. Finally, regarding the account profile, natural and legal persons should care for their image and reputation. Take care of the message in the BIO and use a formal photo. When the account is used professionally, it is advisable to use a logo (LP) and a formal picture (NP) for the BIO. It is also advisable to look for a formal style in the content of the BIO.

These results are helpful for professionals, practitioners and policymakers as they give clues about the differences in using X. They can be the departure point for looking for solutions on X to cope with this gap and analyze the differences.

Specifically, professionals with accounts should generally behave according to their legal or natural status, even differing by gender in the latter case. Having a presence on social networks for personal and/or professional purposes has become commonplace nowadays. Social networks such as X, like other social networks, favor the possibility of making visible what the user wants and encourage interaction. When using networks for professional purposes, you want your accounts to be successful, meaning that you want the information you transmit on them to impact your followers and other accounts. In the case of professional use of the account, they are expected to want their posts to be seen, read, liked, and/or shared by people. In the public eye, their account identifies their brand image. They are displaying a professional image that can lead people to understand that the author of the account has that style. If you know how to manage a social media account correctly, you are sending a positive message that should lead to a good image of your brand as a professional. The proper management of X accounts can help professionals be successful with them, and our tips can help professionals cope with this issue.

Finally, the results and conclusions obtained can help policymakers, as we have obtained results and conclusions on the concrete use of X through institutional and official accounts, among others.

## CRediT authorship contribution statement

**Marcos Antón-Renart:** Writing – review & editing, Writing – original draft, Visualization, Validation, Supervision, Software, Resources, Project administration, Methodology, Investigation, Formal analysis, Data curation, Conceptualization. **Esther Ortiz-Martínez:** Writing – review & editing, Writing – original draft, Visualization, Validation, Supervision, Software, Resources, Project administration, Methodology, Investigation, Formal analysis, Data curation, Conceptualization. **Salvador Marín-Hernández:** Writing – review & editing, Writing – original draft, Visualization, Validation, Supervision, Software, Resources, Project administration, Methodology, Investigation, Formal analysis, Data curation, Conceptualization.

## Data availability statement

Data will be made available on request.

## Funding

This research did not receive any specific grant from funding agencies in the public, commercial, or not-for-profit sectors.

## Declaration of competing interest

The authors declare that they have no known competing financial interests or personal relationships that could have appeared to influence the work reported in this paper.
